# Real-time quantitative PCR: a reliable molecular diagnostic and follow-up tool for ‘minimal residual disease’ assessment in chronic myeloid leukemia

**DOI:** 10.1042/BSR20180974

**Published:** 2018-10-05

**Authors:** Niyaz A. Azad, Zafar A. Shah, Arshad A. Pandith, Roohi Rasool, Samoon Jeelani

**Affiliations:** 1 Department of Immunology and Molecular Medicine, Sher-i-Kashmir Institute of Medical Sciences, Srinagar, Jammu and Kashmir, India; 2 Advanced Centre for Human Genetics, Sher-i-Kashmir Institute of Medical Sciences, Srinagar, Jammu and Kashmir, India; 3 Department of Clinical Hematology, Sher-i-Kashmir Institute of Medical Sciences, Srinagar, Jammu and Kashmir, India

**Keywords:** Cytogenetics, e13a2, e14a2, q-PCR

## Abstract

Molecular monitoring of BCR-ABL transcript levels by real-time quantitative PCR is increasingly being used to diagnose the disease and assess treatment response in patients with chronic myeloid leukemia (CML). This has become particularly relevant when residual levels of leukemia usually fall below the level of detection by cytogenetic analysis. Forty-two CML patients, including 18 males (42.86%) and 24 females (57.14%) aged 7–75 years, were enlisted for the study and followed-up for the response to imatinib treatment. Patients were subjected to Multiplex RT-PCR (reverse-transcriptase PCR) and were all found to harbor either e13a2 or the e14a2, which could be analyzed by a single Taqman probe based quantitation kit (Geno-Sen’s) to quantitate the BCR-ABL transcript load. The Multiplex RT-PCR and peripheral blood cytogenetics providing specific and sensitive detection of BCR-ABL fusion transcripts and metaphase signal load respectively were used as parallel reference tools to authenticate the q-PCR findings. There was 100% concordance between the multiplex RT-PCR and the q-PCR as every positive RT-PCR assay for a transcript reflected as q-PCR load of above 0% for that transcript. q-PCR also demonstrated a strong Pearson correlation with the cytogenetic response.

## Introduction

Chronic myeloid leukemia (CML), the first neoplasm in humans to be associated with a single specific acquired genetic lesion [1], is one of the best understood myeloproliferative disorders at the molecular level. The disease originates from the transformation of a hematopoietic stem cell with consequent expanding myelopoiesis. The reciprocal t(9;22)(q34;q11) translocation is identified as the initial transforming event in the pathogenesis of CML that yields a truncated chromosome 22 called the Philadelphia (Ph) chromosome [2] harboring the BCR-ABL fusion gene which constitutes the molecular basis of the disease [3].

In the vast majority of CML patients (95%) and approximately one-third of Ph+ALL patients, the *BCR* gene breaks in the 5.8-kb breakpoint cluster region (bcr) spanning exons 12–16 and is termed as the major bcr (M-bcr). As a result of alternative splicing, either b2a2 or b3a2 (also called e13a2 and e14a2, respectively) transcripts are formed. The other two bcrs in the *BCR* gene have also been characterized, which are: the minor-bcr (m-bcr) and micro-bcr (µ-bcr) regions [4].

Hematologic, cytogenetic, and molecular monitoring of CML along with BCR-ABL1 mutational analysis have become integral to the routine management of the disease [5,6]. However, the definition of a molecular response as indicative of a high probability of progression-free survival highlights the relevance of molecular analysis for clinical management as increases in the BCR-ABL level can identify patients as probable candidates for kinase domain mutations that lead to imatinib resistance. Therefore, the real-time PCR based molecular quantitative assays (RQ-PCR or q-PCR) can be used as a screening strategy for mutation analysis. Furthermore, as second-generation kinase inhibitors’ clinical use is on, the molecular response remains a primary end point that determines efficacy.

RQ-PCR or q-PCR [7,8] involves extraction of total RNA from the peripheral blood or bone marrow specimen, reverse-transcription of the mRNA so obtained into cDNA, and quantitative (real-time) co-amplification of the target BCR-ABL cDNA and cDNA of an internal control gene. For quantitative molecular assays, standard curves are constructed by serial dilutions of known amount of cloned plasmid containing the fusion DNA, or from serial dilutions of K562 cells in normal DNA. Nowadays, such material is provided by the easily available Taqman probe based kit formulations.

This work is a first of its kind endeavor in our part of the world called Kashmir (North India), where no such work has been attempted before. As our institute (Sher-i-Kashmir Institute of Medical Sciences (SKIMS)) happens to be the only referral institute for leukemia patients including CML, the primary objective behind our work was to establish the real-time quantitative assay for BCR-ABL transcripts in order to help monitor the treatment of CML patients in our setting.

## Materials and methods

### Patients

Forty-two CML patients including 18 males (42.86%) and 24 females (57.14%) aged 7–75 years, of which 19 cases (45.24%) belonged to age group ≤45 years and the rest 23 (54.76%) were >45 years, were enrolled into our cohort study after their screening from the Departments of Medical Oncology, SKIMS, and Clinical Hematology, SKIMS. Informed consent from each patient as well as the approval from the ‘Institute Ethics Committee’ (IEC) of SKIMS was obtained prior to start of the study. Patients were monitored for response to Imatinib (400 mg/day) and were recruited from October 2013 to November 2014 and followed up till May 2016 at the Department of Immunology and Molecular Medicine, SKIMS. The diagnosis of CML was based on characteristic peripheral blood smear analysis and complete blood profiling along with bone marrow examination findings of the patients.

### Molecular analysis (qualitative)

**Figure 1:**
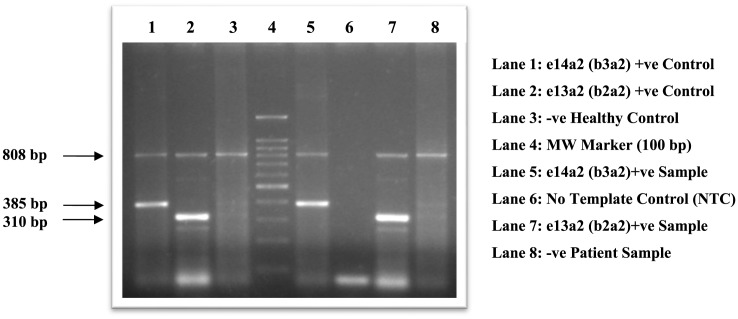
Gel electrophoresis documentation of BCR-ABL fusion gene transcripts in CML

Four milliliters peripheral blood was collected into a lavender-top EDTA vacutainer from each CML patient. Such samples were put to density gradient centrifugation (Ficoll, Sigma) and the white cell component of the peripheral whole blood so obtained was subjected to TRIzol (Amresco) RNA extraction. The extracted RNA was analyzed for purity and integrity by DEPC-treated gel electrophoresis. The RNA was reverse-transcribed by Maxima® cDNA synthesis kit and the cDNA thus obtained was subjected to Multiplex reverse-transcriptase polymerase chain reaction (RT-PCR) or qualitative PCR analysis for fusion gene transcript genotyping (Figure 1). We adopted a multiplex RT-PCR protocol used by Cross et al. [9] to detect three main transcript types of ‘e1a2’, ‘e13a2’, and ‘e14a2’. The primer sequences (Eurofin Oligos) used along with expected transcript amplicons generated are reflected in Table 1. The thermal conditions were used as follows: cyclic denaturation at 94°C for 35 s; cyclic annealing at 61°C for 30 s; cyclic extension at 72°C for 30 s and final extension at 72°C for 7 min. All the cyclic steps in the thermal profile were repeated 35 times. The step of initial denaturation was omitted as cDNA template synthesized as a single strand did not require initial double-strand separation so crucial for DNA, which ruled out any co-amplification of any contaminating DNA sequences due to possible mispriming.

**Table 1:** Primer sequences used in RT-PCR of BCR-ABL transcripts along with transcript amplicons generated

**Primer sequences**		
BCR-C (Forward): 5′-ACCGCATGTTCCGGGACAAAAG-3′		
B2B (Forward): 5′-ACAGAATTCCGCTGACCATCAATAAG-3′		
C5e (Reverse): 5′-ATAGGATCCTTTGCAACCGGGTCTGAA-3′		
CA3 (Reverse): 5′-TGTTGACTGGCGTGATGTAGTTGCTTGG-3′		
**Transcript**	**Primers**	**Amplicon size (bp)**
Normal BCR	B2B+C5e	808
e1a2	BCR-C+CA3	481
e13a2 (b2a2)	B2B+CA3	310
e14a2 (b3a2)	B2B+CA3	385

### Molecular analysis (quantitative)

The RNA samples after normalization to the concentration of approximately 500 ng were subjected to the integrated cDNA synthesis and real-time amplification for the fusion transcript load estimation at baseline and follow-up durations of 3 and 6 months, and 1 year using Taqman probe based BCR-ABL transcript quantitation kit (Geno-Sen’s Genome Diagnostics Pvt. Ltd.) on the Agilent Stratagene Mx-3000-P real-time PCR platform. The value of BCR-ABL transcript was extrapolated from the standard curve and expressed as a normalized ratio of the BCR-ABL transcript to the control ABL-gene transcript.

### Cytogenetic evaluation

**Figure 2:**
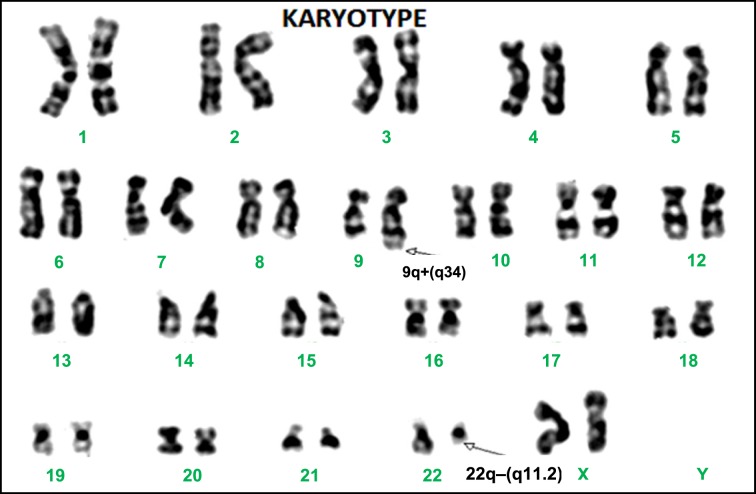
Karyotype showing metaphase spread with a t(9;22)(q34;q11.2) or Ph translocation

Two milliliters of peripheral blood was drawn into green-top heparinized vacutainer from each CML patient for cytogenetic monitoring at 3 and 6 months, and 1 year of imatinib therapy as per the cytogenetic analysis protocol followed by us in our earlier study [10]. Peripheral blood karyotypes were obtained from cultures of such samples, initiated in duplicates as 1 ml each of peripheral blood was inoculated in 5 ml each of RPMI 1640 (Cell Clone) culture medium with 10% FBS (Gibco) at 37°C for 48–72 h. Cell cultures were treated with Colchicine (Loba Chemie, 1 mg/10 ml) along with Ethidium Bromide (1 mg/10 ml) in the final hour of incubation. Cells were subsequently harvested by subjecting them to hypotonic shock with 0.075 M potassium chloride and fixed in 3:1 proportion of methanol and acetic acid. GTG banding was performed as described by Seabright [11] and chromosomes were identified and arranged according to the International System for Human Cytogenetic Nomenclature (ISCN) [12] with the help of a computerized work station – ‘Cytovision’. The number of cells or metaphase spreads investigated for each patient at each analysis ranged from 20 to 30. A representative karyotype with translocation t(9;22)(q34;q11.2), a hallmark in CML, is shown in Figure 2.

## Results

There was 100% concordance between the multiplex RT-PCR and the q-PCR as every transcript load above 0% reflected as positive (+ve) RT-PCR assay for that transcript. At baseline q-PCR analysis, there were numerically more males and patients with e14a2 transcript falling within the load range of ‘40–80%’. More females fell within the range of ‘>80%’ compared to the men patients falling in the range ‘40–80%’ and none of the patients having transcript e13a2 had ‘>80%’ transcript load. The indicated patient parameters of gender, age, and transcript type did not show any significant association with any of the indicated baseline transcript load ranges of ‘0–40%’, ’40–80%’, and ‘>80%’ (Table 2).

**Table 2:** Distribution of baseline transcript load ranges against indicated patient parameters

Analysis	Patients parameters (*n*=42)		Total	Quant. mol. response-transcript load			*P*-value
				0–40%	40–80%	>80%	
Baseline q-PCR	Gender	Males	18	06	11	01	0.6
		Females	24	05	09	10	
	Age	≤45	19	05	10	04	0.8
		>45	23	06	10	07	
	BCR-ABL transcript	e13a2	11	05	06	00	0.7
		e14a2	31	06	14	11	

Most of the patients responded positively to the imatinib therapy and showed clinical improvement registering improved cytogenetic and molecular responses across all the three follow-ups at 3 and 6 months, and 1 year. This helped us correlate the q-PCR analysis in terms of the log reductions in the transcript loads with cytogenetic responses.

As per the European Leukemia Net (ELN) 2013 recommendations for optimal treatment response [13], the 1 log reduction (BCR-ABL transcript ≤10%) is usually coincident with a PCR: 1–35% Ph+ve cells at 3 months. Similarly 2 log reduction (BCR-ABL transcript <1%) and 3 log reduction (BCR-ABL transcript ≤0.1%) coincident with the CCR (complete cytogenetic response: 0% or no Ph+ve cells) from 6 months onward.

Our q-PCR analysis worked out in congruence with the ELN 2013 recommendations [13], which were further fully corroborated by the multiplex RT-PCR findings. At 3 months, we found that the majority of patients with ‘1 log reduction’ in their respective transcript loads were showing PCR and those with ‘no 1 log reduction’ were showing PCR as well as NCR (no cytogenetic response: >95% Ph+ve cells). Besides, the ‘1 log reduction’ seemed to be unrelated to the gender, age, or transcript genotype distribution amongst patients with no statistical significance apparent thereof (Table 3). Similarly, the majority of patients with ‘2 log reduction’ at 6 months demonstrated as CCR cases and patients having ‘no 2 log reduction’ showed up as NCR cases (Table 4). There was no gender, age, or transcript type based association noted. The scenario of ‘3 log reduction’ harmoniously corresponded with the cytogenetic results at 1 year, wherein patients registering ‘3 log reduction’ showed CCR and those showing lesser log reduction were still showing only PCR, which pointed toward their impaired or suboptimal response to treatment [13]. Again, there was no gender, age, or transcript type based association noted (Table 5).

**Table 3:** Distribution of 1 log q-PCR load reduction in terms of different cytogenetic responses at 3 months

qPCR at 3 months (1 log reduction)	Patient parameters (*n=*42)		Total	Cytogenetic response			*P-*value
				CCR	PCR	NCR	
Yes	Gender	Males	14	04	10	0	0.546
		Females	16	02	14	0	
	Age	≤45	13	04	09	0	0.436
		>45	17	02	15	0	
	BCR-ABL transcript	e13a2	10	01	09	0	0.625
		e14a2	20	05	15	0	
No	Gender	Males	04	0	02	02	1.0
		Females	08	0	04	04	
	Age	≤45	06	0	02	04	0.514
		>45	06	0	04	02	
	BCR-ABL transcript	e13a2	01	0	0	01	0.579
		e14a2	11	0	06	05	

1 log reduction: BCR-ABL transcript ≤ 10%

**Table 4:** Distribution of 2 log q-PCR load reduction in terms of different cytogenetic responses at 6 months

qPCR at 6 months (2 log reduction)	Patient parameters (*n=*42)		Total	Cytogenetic response			*P*-value
				CCR	PCR	NCR	
Yes	Gender	Males	16	16	0	0	0.647
		Females	19	18	01	0	
	Age	≤45	15	15	0	0	0.680
		>45	20	19	01	0	
	BCR-ABL transcript	e13a2	10	10	0	0	0.814
		e14a2	25	24	01	0	
No	Gender	Males	02	0	01	01	0.644
		Females	05	01	01	03	
	Age	≤45	04	0	02	02	0.232
		>45	03	01	0	02	
	BCR-ABL transcript	e13a2	01	0	0	01	0.644
		e14a2	06	01	02	03	

2 log reduction: BCR-ABL transcript < 1%.

**Table 5:** Distribution of 3 log q-PCR load reduction in terms of different cytogenetic responses at 1 year

qPCR at 1 year (3 log reduction)	Patient parameters (*n=*42)		Total	Cytogenetic response			*P-*value
				CCR	PCR	NCR	
Yes	Gender	Males	16	16	0	0	1.0
		Females	19	19	0	0	
	Age	≤45	16	16	0	0	1.0
		>45	19	19	0	0	
	BCR-ABL transcript	e13a2	10	10	0	0	1.0
		e14a2	25	25	0	0	
No	Gender	Males	02	0	02	0	0.571
		Females	05	02	03	0	
	Age	≤45	03	01	02	0	0.970
		>45	04	01	03	0	
	BCR-ABL transcript	e13a2	01	0	01	0	0.790
		e14a2	06	02	04	0	

3 log reduction: BCR-ABL transcript ≤ 0.1.

**Figure 3:**
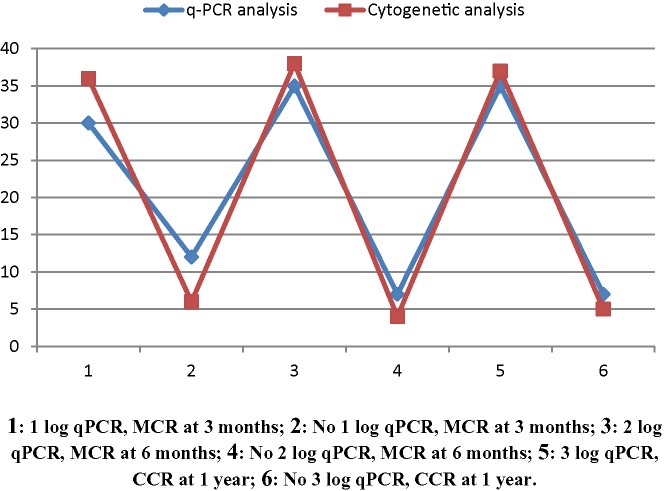
Line plot of corresponding molecular and cytogenetic responses at 3 and 6 months, and 1 year

The overall distribution of patients in terms of q-PCR and cytogenetics is given in table below (Table 6) along with Pearson correlation (Table 7) showing highly significant correlation between the two (*P*<0.0001). A line plot (Figure 3) graphically represents the said correlation, wherein the two lines for molecular and cytogenetic responses seem to deviate slightly at first follow-up of 3 months with successive follow-up of 6 months and 1 year showing enhanced correspondence between the two. This can be explained as due to the fact that initially several patients registering ‘partial cytogenetic response’ had not yet achieved a 1 log molecular response (≤10% BCR-ABL transcript). As the follow-up duration increased, the cytogenetic response reached its limit as ‘complete cytogenetic response’ at 1 year and any further clinical improvement could only be followed through molecular response thereafter.

**Table 6:** Corresponding molecular and cytogenetic responses at 3 and 6 months, and 1 year

**Molecular response (q-PCR)**					
At 3 months		At 6 months		At 1 year	
1 Log	No 1 Log	2 Log	No 2 Log	3 Log	No 3 Log
30	12	35	07	35	07
**Cytogenetic response**					
At 3 months		At 6 months		At 1 year	
MCR	No MCR	MCR	No MCR	CCR	No CCR
36	06	38	04	37	05

Abbreviation: MCR, major cytogenetic response (a combination of PCR and CCR).

**Table 7:** Statistical correlation between molecular and cytogenetic responses at 3 and 6 months, and 1 year

**Correlation matrix (Pearson)**		
Variables	Molecular response	Cytogenetic response
Molecular response	**1**	**0.989**
Cytogenetic response	**0.989**	**1**
Values in bold are different from ‘0’ with a significance level α = 0.05		
**Coefficients of determination (*R^2^*)**		
Variables	Molecular response	Cytogenetic response
Molecular response	**1**	0.979
Cytogenetic response	0.979	**1**
***P*-values**		
Variables	Molecular response	Cytogenetic response
Molecular response	**0**	**0.000**
Cytogenetic response	**0.000**	**0**
Values in bold are different from ‘0’ with a significance level ***α*** = 0.05		

## Discussion

Even though the establishment of CCR as indicative of the complete eradication of cells harboring the Ph chromosome is considered a significant milestone in the treatment of CML, the ultimate goal of therapy for CML remains getting patients rid of the molecular counterpart of the Ph chromosome, the BCR-ABL fusion gene transcript, as patients achieving CCR may still harbor up to 10^9^ leukemic cells in their bodies [14]. Moreover, early reduction in BCR-ABL transcript levels predicts cytogenetic response in chronic phase CML patients treated with imatinib and the reduction in BCR-ABL correlates with prognosis [15–17].

Over the past many years, several groups have developed quantitative PCR or q-PCR assays to measure BCR-ABL transcript levels in the blood and marrow that enable the dynamics of residual disease to be monitored over time, thereby providing a viable alternative for disease monitoring [18,19]. The transcript level correlates with the number of leukemic cells present in the blood and marrow and can be used as an accurate barometer of the response to therapy [20].

To establish the hitherto unavailable real-time PCR (q-PCR) assay here in Kashmir (North India) and evaluate it as a detection and quantitative follow-up tool for BCR-ABL fusion transcripts, we performed side-by-side analysis with conventional RT-PCR for BCR-ABL transcripts as well as peripheral blood cytogenetics (PBC) to draw an overall comparative picture. The clinical utility of the technique was investigated in terms of the assessment of the molecular residual disease after imatinib therapy of Ph-positive CML patients. All the 42 patients reporting +ve for RT-PCR were detected by q-PCR as well with 100% concordance rate between them. This is higher than 96.3% reported by Lee et al. [21].

For the follow-up of CML patients, the ELN recommendations [13] require molecular response monitoring every 3 months until a major molecular response (MMR) is at least achieved, then every 3–6 months. BCR-ABL transcript levels ≤10% at 3 months, <1% at 6 months, and ≤0.1% from 12 months onward define optimal response, whereas >10% at 6 months and >1% from 12 months onward define failure. Similarly, PCR at 3 months and CCR from 6 months onward define optimal response, whereas NCR at 3 months, less than PCR at 6 months and less than CCR from 12 months onward define failure. Between the optimal response and failure, there is an intermediate warning zone that calls for more frequent monitoring.

Keeping in view the said criteria, we correlated the cytogenetic analysis with the molecular (q-PCR) analysis and found that the two analyses helped us categorize the patients as imatinib responders and imatinib-resistant cases appropriately and the correlation happened to be highly significant (*P*<0.001). This was similar to that reported by others [21], reiterating the fact that levels of BCR-ABL transcript in the peripheral blood by q-PCR show excellent congruity with those of metaphase cytogenetics [22] and buttresses the assessment that q-PCR is a reliable minimal residual disease assessment tool. Similarly, the real-time quantitative PCR analysis of patients treated with imatinib has shown a strong correlation between the percentage of Ph-positive metaphases and simultaneous study of peripheral blood BCR-ABL levels measured by q-PCR [23,24].

In our assay, we used TaqMan chemistry-based kit with *ABL* as the control gene on Agilent Stratagene Mx 3000P real-time PCR platform. As a matter of fact, the q-PCR results may vary with respect to the type of instrument used, the primer and probe location, the real-time chemistry, and the control gene employed [25,26] or due to inter-lab differences in sample collection, storage, processing, RNA integrity etc. [27], thereby leading to variation in the sensitivity and hence measurement reliability. It is therefore essential that each laboratory establishes the limits for their method to allow accurate interpretation of serial monitoring and the estimation of measurement reliability. The appropriate quality assurance as per the international standards is an important aspect of the development of any method used to monitor patients. However, for the measurement of BCR-ABL transcripts by quantitative PCR, use of certified international reference and control materials make the assay a somewhat uneconomical prospect for medical facilities like ours having budgetary constraints. To address this issue, we established the real-time quantitative molecular assay (q-PCR) in conjunction with the Multiplex RT-PCR and the cytogenetic evaluation as explained here above for a reliable clinical management of CML until further improving it in-line with the international standardization, which anyway remains a logistical and fiscal challenge for many labs the world over including United States [27].

## Conclusion

We established the Taqman probe based real-time quantitative molecular assay (q-PCR) for CML patients in Kashmir (North India) at SKIMS and found it as a reliable molecular diagnostic and follow-up analytical tool in the disease.

## Abbreviations

bcr: breakpoint cluster region
CCR: complete cytogenetic response
CML: chronic myeloid leukemia
ELN: European Leukemia Net
NCR: no cytogenetic response
PCR: partial cytogenetic response
Ph: Philadelphia
RQ-PCR or q-PCR: real-time PCR based molecular quantitative assay
RT-PCR: reverse-transcriptase PCR
SKIMS: Sher-i-Kashmir Institute of Medical Sciences

## References

[1] Nowell P.C. and Hungerford D.A. (1960) A minute chromosome in human chronic granulocytic leukemia. Science 132, 1497 10.1126/science.144.3623.1229 14150328

[2] Rowley J.D. (1973) A new consistent chromosomal abnormality in chronic myelogenous leukemia identified by Quinacrine fluorescence and Giemsa staining. Nature 243, 290–293 10.1038/243290a0 4126434

[3] Shtivelman E., Lifshitz B., Gale R.P. and Canaani E. (1985) Fused transcripts of abl and bcr genes in chronic myelogenous leukaemia. Nature 315, 550–554 10.1038/315550a0 2989692

[4] Melo J.V. (1996) The diversity of BCR-ABL fusion proteins and their relationship to leukemia phenotype. Blood 88, 2375–2384 8839828

[5] O’Brien S.G., Guilhot F., Larson R.A. (2003) Imatinib compared with interferon and low-dose cytarabine for newly diagnosed chronic-phase chronic myeloid leukemia. N. Engl. J. Med. 348, 994–1004 10.1056/NEJMoa022457 12637609

[6] Baccarani M., Saglio G., Goldman J. (2006) Evolving concepts in the management of chronic myeloid leukemia: recommendations from an expert panel on behalf of the European Leukemia Net. Blood 108, 1809–1820 10.1182/blood-2006-02-005686 16709930

[7] Higuchi R., Dollinger G., Walsh P.S. and Griffith R. (1992) Simultaneous amplification and detection of specific DNA sequences. Biotechnology 10, 413–417 10.1038/nbt0492-413 1368485

[8] Higuchi R., Fockler C., Dollinger G. and Watson R. (1993) Kinetic PCR: real time monitoring of DNA amplification reactions. Biotechnology 11, 1026–1030 7764001 10.1038/nbt0993-1026

[9] Cross N., Melo J., Feng L. and Goldman J. (1994) An optimized multiplex polymerase chain reaction (PCR) for detection of BCR-ABL fusion mRNA in haematological disorders. Leukemia 8, 186–189 8289486

[10] Azad N.A., Baba S.M., Shah Z.A. (2015) Phytohemagglutinin-induced peripheral blood cytogenetics: a valid means for diagnosis and imatinib therapy monitoring of chronic phase chronic myeloid leukemia patients. J. Cancer Sci. Ther. 7, 242–248

[11] Seabright M. (1971) A rapid banding technique for human chromosomes. Lancet 2, 971–972 10.1016/S0140-6736(71)90287-X 4107917

[12] Mitelman F. (1995) ISCN 1995: An International System for Human Cytogenetic Nomenclature, S. Karger, Basel

[13] Baccarani M., Deininger M.W., Rosti G. (2013) European LeukaemiaNet recommendations for the management of chronic myeloid leukaemia. Blood 122, 872–884 10.1182/blood-2013-05-501569 23803709 PMC4915804

[14] Lowenberg B. (2003) Minimal residual disease in chronic myeloid leukemia. N. Engl. J. Med. 349, 1399–1401 10.1056/NEJMp038130 14534331

[15] Branford S., Rudzki Z., Harper A. (2003) Imatinib produces significantly superior molecular responses compared to interferon α plus cytarabine in patients with newly diagnosed chronic myeloid leukemia in chronic phase. Leukemia 17, 2401–2409 10.1038/sj.leu.2403158 14523461

[16] Merx K., Muller M.C., Kreil S. (2002) Early reduction of BCR–ABL mRNA transcript levels predicts cytogenetic response in chronic phase CML patients treated with imatinib after failure of interferon α. Leukemia 16, 1579–1583 10.1038/sj.leu.2402680 12200666

[17] Wang L., Pearson K., Ferguson J.E. (2003) The early molecular response to imatinib predicts cytogenetic and clinical outcome in chronic myeloid leukaemia. Br. J. Haematol. 120, 990–999 10.1046/j.1365-2141.2003.04200.x 12648069

[18] Lion T., Izraeli S., Henn T. (1992) Monitoring of residual disease in chronic myelogenous leukemia by quantitative polymerase chain reaction. Leukemia 6, 495–499 1602787

[19] Lin F., van Rhee F., Goldman J.M. (1996) Kinetics of increasing BCR–ABL transcript numbers in chronic myeloid leukemia patients who relapse after bone marrow transplantation. Blood 87, 4473–4478 8639810

[20] Branford S., Hughes T.P. and Rudzki Z. (1999) Monitoringchronicmyeloid leukaemia therapy by real-time quantitative PCR in blood is a reliable alternative to bone marrow cytogenetics. Br. J. Haematol. 107, 587–599 10.1046/j.1365-2141.1999.01749.x 10583264

[21] Lee W.I., Kantarjian H., Glassman A., Talpaz M. and Lee M.S. (2002) Quantitative measurement of BCR/abl transcripts using real-time polymerase chain reaction. Ann. Oncol. 13, 781–788 10.1093/annonc/mdf156 12075749

[22] Fan H. and Robetorye R.S. (2010) Real-time quantitative reverse transcriptase polymerase chain reaction. Methods Mol. Biol. 630, 199–213 10.1007/978-1-60761-629-0_13 20300999

[23] Muller M.C., Gattermann N., Lahaye T. (2003) Dynamics of BCR–ABL mRNA expression in first-line therapy of chronic myelogenous leukemia patients with imatinib or interferon α/ara-C. Leukemia 17, 2392–2400 10.1038/sj.leu.2403157 14523462

[24] Hughes T. and Branford S. (2003) Molecular monitoring of chronic myeloid leukemia. Semin. Hematol. 40, 62–68 10.1053/shem.2003.50044 12783378

[25] Emig M., Saussele S., Wittor H. (1999) Accurate and rapid analysis of residual disease in patients with CML using specific fluorescent hybridization probes for real time quantitative RT-PCR. Leukemia 13, 1825–1832 10.1038/sj.leu.2401566 10557058

[26] Radich J.P., Gooley T., Bryant E. (2001) The significance of bcr-abl molecular detection in chronic myeloid leukemia patients ‘late, 18 months or more after transplantation. Blood 98, 1701–1707 10.1182/blood.V98.6.1701 11535500

[27] Arora R. and Press R.D. (2017) Measurement of BCR-ABL1 transcripts on the International Scale in the United States: current status and best practices. Leuk. Lymphoma 58, 8–16 10.1080/10428194.2016.1190974 27412040

